# Efficacy Evaluation of a non-contact automatic articulating paper dispenser in controlling articulating paper microbial contamination

**DOI:** 10.1038/srep46729

**Published:** 2017-05-03

**Authors:** Yajin Li, Litong Chen, Fusong Yuan, Yugui Li, Yongsheng Zhou, Yuchun Sun

**Affiliations:** 1 Department of Prosthodontics, Peking University School and hospital of Stomatology, Beijing, China; 2 Center of Digital Dentistry, Faculty of Prosthodontics, Peking University School and hospital of Stomatology & National Engineering Laboratory for Digital and material technology of stomatology & Research Center of Engineering and Technology for Digital Dentistry, Ministry of Health & Beijing Key Laboratory of Digital Stomatology, Beijing, China

## Abstract

This study is to quantitatively evaluate the efficacy of using a non-contact automatic articulating paper dispenser for reducing microbial articulating paper contamination. Articulating papers in four-handed mode, non-four-handed mode, and via an automatic articulating paper dispenser were evaluated. An adenosine triphosphate bioluminescence assay was used to quantitatively measure the relative light unit (RLU) values of the rest unused articulating papers in the same package to detect contamination at 4 time points, and triplicate examinations were performed for all three methods. The RLUs were recorded, compared, and evaluated. For four-handed mode (n = 36), the RLUs at the four time points were 2.44, 32.89, 37.89, and 27.22, with a satisfactory rate of 94%. The RLUs for non-four-handed mode (n = 36) were 2.22, 286.44, 299.44, and 493.56, with a satisfactory rate of 36%. The RLUs for using the automatic dispenser (n = 36) were all 0 with a satisfactory rate of 100%. The satisfactory rates were significantly different among three methods. No significant differences were observed in the satisfactory rates for the four time points samples. Contact by gloved hands can cause severe biological contamination of articulating paper. However, by using standard four-handed mode or a non-contact automatic articulating paper dispenser, contamination can be controlled.

Articulating paper is an essential and consumable material that is widely used as a diagnostic tool for occlusal analysis in many fields of dental practice, including prosthodontics, orthodontics, endodontics, and periodontics. Articulating papers of various thicknesses and colors are used to provide diagnostic information on the occlusal status between the maxillary and mandibular teeth of patients^1^. In particular, articulating papers are frequently and extensively used in prosthodontics during the fitting of prosthetic teeth or dentures. They are usually packaged and sold in books that contain a dozen to hundreds of sheets which can be used for several patients. Thus, in clinical practice, direct contact with the articulating paper when manually extracting a sheet from a book can cause contamination. Repeated manual extraction can lead to contamination of the remaining sheets and the package, which can become a source of cross-infection. Because of its special composition, articulating paper cannot be disinfected using conventional techniques such as high temperature and high pressure sterilization methods-the use of these measures will compromise its efficacy.

There are mainly two methods of accessing articulating paper in various dental clinics. One common method is the standard four-handed mode (a nurse extracts articulating paper from the package and passes to the dentist with clean hands or wearing sterile gloves), which can effectively reduce cross-infection risks but at the cost of heavier manpower. The other method frequently observed in some basic-level hospitals or by intern is the non-four-handed mode (a dentist extracts articulating paper from the package with gloved hands), which does not conform to the international standard of infection control and leads to high cross-infection risks^2^. With the rapid development from traditional prosthesis to implantation, active hemorrhage possibility during denture fitting increases and cross-infection risk via articulating paper surges. However, cross-infection risks via articulating paper contamination resulted from both modes have not been quantitatively evaluated by literature.

According to the clinical problems mentioned above, we have developed a non-contact automatic articulating paper dispenser, theoretically to eradicate cross-infection risks via articulating paper contamination from operation by hand. Therefore, this study set the four-handed mode as the main control group and quantitatively evaluated the efficacy of the non-contact automatic articulating paper dispenser in controlling the articulating paper microbial contamination; meanwhile, evaluated the articulating paper microbial contamination via non-four-handed mode as references to management and policy making for clinical infection control.

## Results

### Average relative light unit readings

**Figure 1:**
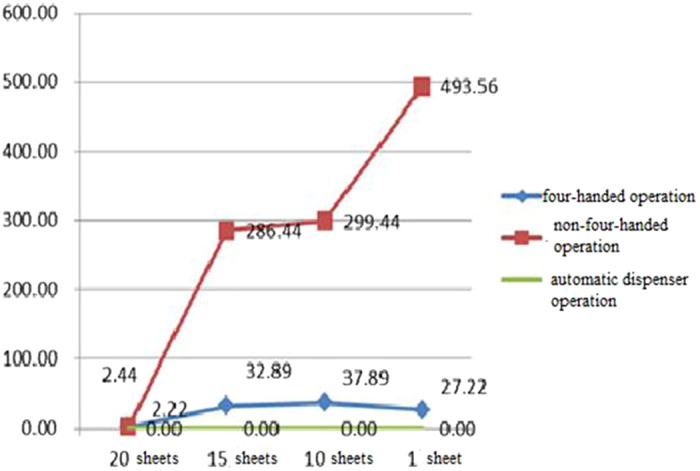
The average relative light units of the samples at various time points for the four-handed mode group, non-four-handed mode group, and the automatic dispenser mode group.

The RLUs of the samples in four-handed mode (RLU = 2.44) and non-four-handed mode (RLU = 2.22) were within safe ranges for the unused articulating paper. For the books with five used sheets, 10 used sheets, and 19 used sheets, RLUs of the rest unused samples in four-handed mode were at 32.89, 37.89, and 27.22, respectively, with the highest value of 140. The RLUs for non-four-handed mode were 268.44, 299.44, and 249.56, respectively, with the highest value of 1,680, which was 16 times above the safety threshold. Lastly, for using the automatic dispenser, the average RLUs were 0, 0, 0, and 0, respectively (Fig. 1).

### Comparison of the satisfactory rate

The satisfactory rates among the different operations are listed in Table 1. Two samples from the four-handed mode group (n = 36) were unsatisfactory, resulting in a 94% satisfactory rate. And 23 samples from the non-four-handed mode group (n = 36) were unsatisfactory, resulting in a satisfactory rate of 36%. When using the automatic dispenser (n = 36), the satisfactory rate reached 100%. Statistical significances were observed in the samples among the groups (P < 0.01).

No significant differences were observed in the satisfactory rates among the four time points. After a new package of articulating paper began to be used, the satisfactory rates were 30% for the 15 remaining articulating paper sheets, 30% for 10 remaining sheets, and 33% for 1 remaining sheet (P = 0.944) (Table 2).

## Discussion

Dental procedures are manually performed by dentists or nurses within the patient’s mouth, which generally contains saliva, blood, and a variety of microorganisms. In clinical practice, it is difficult to determine whether the patient is a carrier of hepatitis B, acquired immune deficiency syndrome, or other infectious diseases^3^. A randomized study of bacteriological monitoring was conducted to evaluate the hygienic status of the hands of dental personnel when performing dental procedures. The results showed a low satisfactory rate that did not fulfill the health standards^4^. In this study, the main control group strictly follow infection control regulations and applied standard four-handed mode to accessing articulating paper, but non-four-handed mode to accessing articulating paper exists in actual clinical situation (which is hard to be eliminated in a short term or be replaced promptly), and we also quantitatively evaluated the cross-infection risks via accessing articulating paper in non-four-handed mode, expecting to provide a quantitative reference for clinical infection control. For dental restoration, articulating paper is routinely accessed from the dental bracket via four-handed mode or non-four-handed mode. Cross-infection risks can be in both modes via articulating paper contamination. In the four-handed mode operation, a nurse extracts the articulating paper sheets from the package with clean hands or sterile gloves. Contamination and cross-infection risks may exist when the nurses commit a non-standard operation due to subjective factors, limited clinical experiences and incidental factors. The non-four-handed mode, applied in some basic-level hospitals and the dental chairs for the interns leads to a higher cross-infection risk without nurse’s assisting, the dentist usually has to extract the articulating paper with contaminated gloved hands. Thus, contamination to the exterior package of the articulating paper or the surface of the remaining sheets in the package may thus transmit pathogenic organisms between patients. In the present study, we noted that repeated manual operations during dental restoration and denture fitting procedures usually led to contamination of the remaining articulating paper in the package by direct contact with the contaminated hands of the dental personnel. However, by using a non-contact automatic articulating paper dispenser, direct contact of contaminated gloved hands with the remaining sheets can be effectively avoided, thus the cross-infection risk can be reduced.

We used ATP bioluminescence analysis, which enabled rapid detection of microbial contamination. ATP is a universal energy molecule found in all living organisms including microorganisms. The presence of ATP indicates the occurrence of microbial contamination or the presence of a food, drink, or body fluid that can harbor microorganisms^5^. The presence of a large amount of ATP on a clean and disinfected surface indicates failure of the cleaning method, which is associated with the risk of infection^6^. Because it is simple to use, lightweight, and reliable, the ATP bioluminescence swabbing technique is significantly superior to the conventional bacterial colony counting method, which involves professional laboratory examinations. Thus, ATP bioluminescence analysis has been widely used for monitoring and controlling infections in healthcare institutions (e.g., monitoring hygiene during dental procedures and detecting the contamination status and cleanliness of surfaces in dental clinics)^7^,^8^,^9^,^10^. In the present study, the ATP bioluminescence assay was used to reflect the contamination status of a surface with dental material. However, this method alone can only measure the quantity of organisms and cannot identify the type of organisms. A study reported that ATP bioluminescence assay, which is influenced by organic subjects, exfoliated cells, and other factors, cannot determine whether the contamination is a pathogenic microorganism, and it cannot qualitatively assess the contamination^10^. The type of bacteria, viruses, and other pathogens on the surface of articulating paper is directly associated with the risk of the resulting cross-infection. Hence, further research is needed to investigate the type and quantity of pathogens on the surface of articulating paper at various time points and on the exterior surface of its package.

Table 1 showed that 34 samples in the four-handed mode group (n = 36) were satisfactory, and only two RLUs exceeded the threshold. The reason for the high satisfactory rate is that the operators in this group followed the professional operational procedures for dental restoration. Articulating paper was pre-prepared before the denture fitting procedure. Because of diverse patient conditions, the quantity of articulating paper sheets required for each treatment was difficult to predict, which often lead to an insufficient number of sheets during the denture fitting procedures. The extraction of articulating paper either with bare hands to avoid contamination from contaminated gloves or with sterile gloves can acquire satisfactory results. The data of the present study displayed that microbial contamination were detected in the remaining articulating paper even in four-handed mode while none by using a non-contact automatic articulating paper dispenser. This data hint that compared to four-handed mode, the non-contact automatic articulating paper dispenser can thoroughly avoid contamination via articulating paper and possible cross-infection risks. In addition, we found that most samples in the four-handed mode were satisfactory, two samples were unsatisfactory. The nurses in both cases reported that the pre-prepared articulating paper was insufficient for the procedures. Thus, the contamination may have been caused either by direct contact with the articulating paper by the dentists’ hands without the nurses’ assistance due to a high patient volume or by the nurses’ incidental non-standard operation of extracting extra articulating sheets. However, the samples from the non-four-handed mode group indicated worse findings. Various degrees of contaminations were detected in the remaining articulating paper. The highest RLU was 16 times above the threshold. The cross-infection risk is high if the next patient uses the contaminated articulating paper. This may be attributed to the fact that the dental students directly accessed the articulating paper during the fitting procedures. Thus, we believe that the contamination of articulating paper is controllable when the conditions allow for it. Both four-handed mode and application of a non-contact automatic articulating paper dispenser can reduce the microbial contamination below threshold, while the latter can eradicate such contamination.

In addition, repeated access to the articulating paper from a book of sheets by various operators is theoretically hypothesized to increase the degree of biological contamination. However, Table 2 showed that RLUs of the remaining articulating paper at various time points were not significantly increased. The underlying mechanism for this may be explained by the fact that multiple operations can only increase the possibility of repeated contamination at the exterior surface of the package while sparing the remaining sheets inside the package. Therefore, proper four-handed mode is required to resolve the issue of cross-infection caused by multiple accesses to the articulating paper. In dental clinics where conditions do not allow four-handed mode, proper operational guidelines should be strictly followed, and a specific device should be used to eradicate microbial contamination via the articulating paper.

In clinical practice, we recommend the non-contact automatic articulating paper dispenser in the dental diagnosis and treatment unit, it quickly and efficiently avoids cross-infection risk caused by traditional way to access the articulating paper without increasing manpower and relevant cost. Moreover, this type of operation can reduce time taken by dentist-nurse communication and decrease chair-side treatment duration, and reduce the workload for dental assistant and improve work efficiency. In addition, using the non-contact automatic articulating paper dispenser will avoid operator’s subjective factors, limited clinical experiences and incidental factors leading to accidental non-standard operation and thus reduce relevant contamination and cross-infection risks. At the same time, the constitution and thickness of carbon blades and surface cleaning method of the equipment will influence the using result. Because the constitution and thickness interfere with ribbon contamination lying in the thickness and angle of the blades, in order to interfere with ribbon contamination, the design of these items is the same as surgical blades. The thickness of the blades is 0.5 mm; the angle of the blades is 20~26°. And regular surface cleaning by Sterile Carbasus dipped in 75% medical alcohol can reduce the contamination index and influence experiment result because bacteria may attach to the surface of the equipment during its operation since the clinical environment is full of bacteria.

Direct contact with the articulating paper using gloved hands during dental procedures can cause severe biological contamination. However, by accessing articulating paper using standard four-handed mode or via an automatic articulating paper dispenser the amount of microorganisms on the articulating paper’s surface can be controlled at a safe level. In addition, multiple accesses to the articulating paper did not significantly increase microbial contamination on the surface of the remaining sheets.

## Materials and Methods

### Materials

Articulating paper is most commonly packaged as a book of 20 red sheets (30 μm thin) for clinics (Shenyang Ke Xing Model & Material Co., Ltd., Shenyang, China).

To measure the hygienic status of hands and the surfaces of objects, we used the System SURE PLUS ATP luminometer, along with standardized reagents and sampling swabs included by the manufacturer (Beijing New Century Biochemical Technology Development Co., Ltd., Beijing, China). The system has a detection accuracy of 1 femtomole (1 × 10^−15^ mole ATP) of adenosine triphosphate (ATP). A relative light unit (RLU) ≤ 30 is considered satisfactory for a clean surface. In the present study, a new, unused package was considered as a clean surface. For used packages, an RLU ≤ 100 was considered satisfactory.

### Methods

#### Ethics Statement

The study was approved by the Bioethics Committee of Stomatological Hospital of Peking University, Beijing, China. (No. PKUSSIRB-2012054. Date: 31/10/2012). All the methods in this study were performed in accordance with the relevant guidelines and regulations. We obtained written informed consents from all the participants in our study. And all of these procedures were part of patients’ routine care. Authors anonymized the patient data.

#### Design and prototype of the non-contact automatic articulating paper dispenser

**Figure 2:**
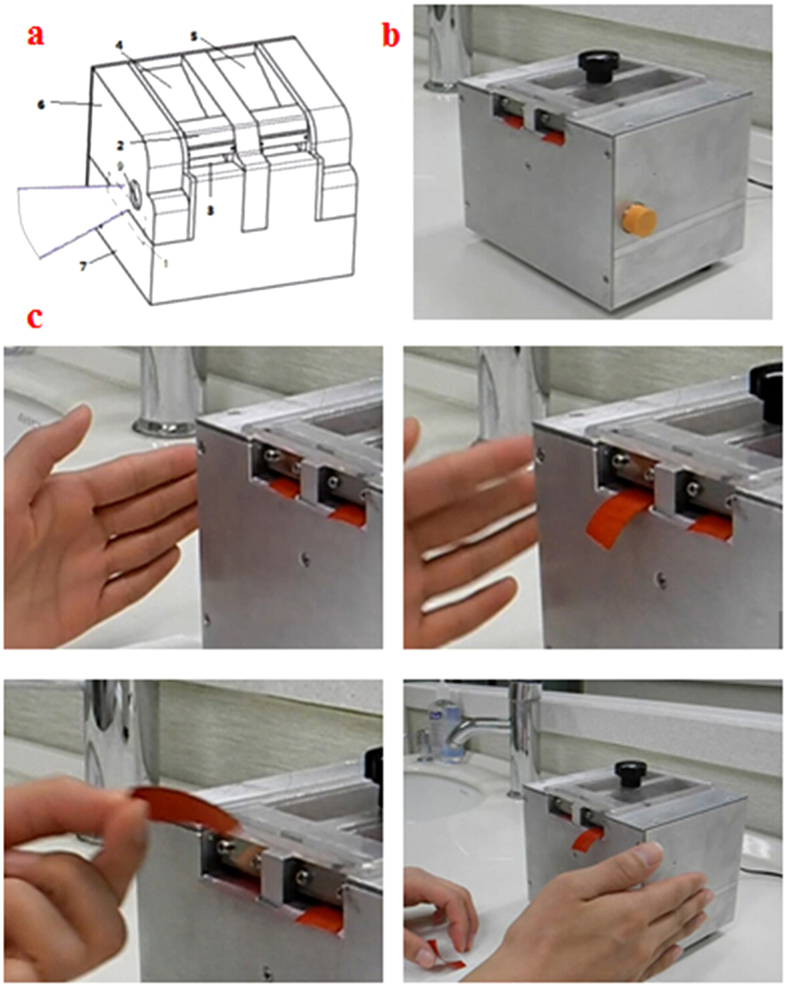
(**a**) The design drawing of non-contact automatic articulating paper dispenser; (**b**) the physical map of non-contact automatic articulating paper dispenser; (**c**) the operating process of non-contact automatic articulating paper dispenser.

The device was equipped with a photoelectric sensor (Fig. 2). Its major components included an articulating paper slot (one and two), a sensor, rollers, isolationhood, rechargeable power supply, relays, motors, exterior cover, dust-proof blade holder, pedestal and carbon blades. The material of carbon blades is 7C27Mo2, the same as surgical blades. Carbon blades must be sterilized by high temperature and pressure before fixed in the dispenser. They should be changed every day. And the specification of the blades is 3(length)*2(width)*0.5 mm (thickness). The exterior cover was mounted on both sides of the pedestal, and the sensor was housed inside the cover and was connected to the small apertures of the cover. Paper slots one and two were mounted on top of the pedestal, and the isolation hood was assembled above the paper slots. The dust-proof blade holder was placed anterior to the paper slots. The sensor was connected to the relay, which was connected to the motor that was connected to the rollers. The rollers were located below the dust-proof blade holder. Articulating paper that was transported by the rollers could be cut by the blades, which were embedded in the dust-proof blade holder. During an operation, the articulating paper was automatically dispensed at a uniform velocity once the operator’s hand was placed within 0–3 cm of the photoelectric sensing area located adjacent to the device. Once a suitable length of paper was dispensed, the hand could move away from the sensing area. The dispensed paper was neatly cut by gently pulling the paper upward, and the process was completed. To enhance its applicability in clinics, the device simultaneously stored articulating papers of two different thicknesses, which were controlled by its sensors on the left and right side (Fig. 1). The surface of the equipment is cleaned by Sterile Carbasus dipped in 75% medical alcohol. And it is cleaned after finishing a patient every time.

#### Clinical operation procedures

Four-handed mode group; In strict accordance with the universal regulations of aseptic techniques during four-handed dentistry.

Preparing for a procedure: pre-determine needs by checking on the patient’s treatment plan and prepare all instruments, equipment, materials and medicaments for each procedure. Leave them bagged until the patient is seated and ready for treatment. Pre-dispense disposables (articulating paper sheets and etc.) in a plastic tray. And place them all on dental bracket table covered by a piece of fresh, clean, disposable plastic drape, and reduce cross-infection risks.

During treatment: Non-disposable and non-sterilizable materials (articulating paper package and etc.) should be placed in clean drawers. A nurse uses sterile gloves or clean hands when extracts extra sheets from articulating paper package and passes to the dentist.

After treatment: A nurse cleans up the dental bracket and replaces all the disposables between patients.

Non-four-handed mode group; Based on the present clinical situation, a nurse’s assisting work is done by the dentist.

Preparing for a procedure: Pre-dispense articulating paper sheets and package in a plastic tray. The package is prepared for spare use during treatment (for example, fitting in restorations). The rest of preparing work is same with the four-handed mode group.

During treatment: If more sheets of articulating paper are needed during treatment, the dentist extracts extra sheets from the package. The rest work is same with the four-handed mode group.

After treatment: Wipe the exterior of articulating paper package with antiseptic wipe and reserve the remaining sheets for next patients. The rest work is same with the four-handed mode group.

Non-contact automatic articulating paper dispenser group; Replace four-handed mode by a non-contact automatic articulating paper dispenser without relevant cross infection risks.

Preparing for a procedure: Pre-dispense a non-contact automatic articulating paper dispenser on dental bracket table covered by a piece of fresh, clean, disposable plastic drape. The rest of preparing work is same with the four-handed mode group.

During treatment: If more sheets of articulating paper are needed during treatment, the dentist extracts extra single sheet from the non-contact automatic articulating paper dispenser, avoiding direct contact of contaminated gloves to the rest articulating paper sheets. The rest work is same with the four-handed mode group.

After treatment: Wipe the exterior of the non-contact automatic articulating paper dispenser with antiseptic wipe and keep the machine in a clean cabinet or drawer for next patients. The rest work is same with the four-handed mode group.

### Measurement

Four-handed mode was performed by one dentist who was at least an associate professor and was assisted by three nurses with at least 2 years of experience. The operators were required to use articulating paper from each book in the same order. The top sheet of the remaining articulating paper was sampled. For each book of articulating paper, the remaining sheets were examined at four time points when various amounts of articulating papers were used. This included an unused book, a book with five sheets used, a book with 10 sheets used, and a book with 19 sheets used, which resulted in a sample set. For each nurse, triplicate examinations of the sample set were performed. Thus, nine groups of examinations were performed, which included 36 samplings for three nurses.

Non-four-handed mode was performed by dentists and nurses at a 3:1 ratio (i.e., three dental interns were assisted by one nurse). Three diagnostic positions were randomly chosen using the same sampling method. Thus, the four-handed and non-four-handed mode conditions yielded a total of 72 samples. We also evaluated the articulating paper used in mode with the non-contact automatic articulating paper dispenser. The articulating paper was accessed as required during treatment and was examined using the same method.

### Sampling of organisms and ATP bioluminescence analysis

ATP bioluminescence swabbing was performed on both sides of each articulating paper and on the two edges of the remaining sheets, as per the manufacturer’s guidelines. The sampling swab was placed back into the test tube after sampling, and it was then snapped, squeezed, and gently shaken 15 times. The test tube was inserted vertically into the System SURE PLUS ATP luminometer to obtain the RLU readings.

### Statistical analysis

We obtained 108 RLU readings for 27 groups of samples, and statistical analysis was performed using SPSS, version 13.0 (IBM Corp., Armonk, NY, USA). The chi-square test was used to compare the satisfactory rates of the samples from the various operations and to compare those from the four time points.

## Additional Information

**How to cite this article:** Li, Y. *et al*. Efficacy Evaluation of a non-contact automatic articulating paper dispenser in controlling articulating paper microbial contamination. *Sci. Rep.* **7**, 46729; doi: 10.1038/srep46729 (2017).

**Publisher's note:** Springer Nature remains neutral with regard to jurisdictional claims in published maps and institutional affiliations.

## Figures and Tables

**Table 1:** Comparison of satisfactory rates of relative light units (RLUs) among the samples from the operation groups.

Results	Number of satisfactory results	Number of unsatisfactory results	Satisfactory rate	*χ*	*P* value
Groups					
Four-handed mode group	34	2	94%		0.00
Non-four-handed mode group	13	23	36%	50.695	
Operation using the non-contact automatic dispenser	36	0	100%		

**Table 2:** Comparison of satisfactory relative light unit (RLU) values among samples at various time points after a new package of articulating paper began to be used.

Results	Number of satisfactory RLUs	Number of unsatisfactory RLUs	Satisfactory rate	*χ*	*P* value
Group					
15 sheets remaining	19	8	30%		
10 sheets remaining	19	8	30%	0.116	0.944
1 sheet remaining	18	9	33%
